# Supplementary material for: Coupled strontium-calcium isotopes in Archean anorthosites reveal a late start for mantle depletion
Source: Nat Commun. 2025 Oct 31;16:9642. doi: 10.1038/s41467-025-64641-2 (PMC12578967; doi:10.1038/s41467-025-64641-2)
Supplement: Supplementary file 1 — Supplementary Information [file 41467_2025_64641_MOESM1_ESM.pdf]

Coupled strontium-calcium isotopes in Archean anorthosites reveal a late start for mantle depletion

Supplementary Information

Matilda Boyce[1*], Anthony Kemp[1], Chris Fisher[1], Dan Bevan[1,2], Aleksey Sadekov[2], Jamie Lewis[3], Simon Wilde[4], Tim Ivanic[5], Tim Elliott[3]

1. School of Earth Sciences, The University of Western Australia, Crawley, WA 6009, Australia
2. Centre for Microscopy, Characterisation and Analysis, The University of Western Australia, Crawley, WA 6009, Australia
3. Bristol Isotope Group, School of Earth Sciences, University of Bristol, BS8 1RJ, UK
4. School of Earth and Planetary Sciences, Curtin University, Bentley, WA 6102, Australia
5. Geological Survey of Western Australia, Department of Energy, Mines, Industry Regulation and Safety, East Perth, WA 6004, Australia

** Corresponding author:* *matilda.boyce@uwa.edu.au*

Supplementary Table 1

Reservoir	Time before present (Ga)	$^{87}Rb/^{86}Sr$ (time integrated)	$^{87}Sr/^{86}Sr$
Solar system (CAI)	4.567	–	0.698975 ± 8 [1]
Bulk Moon (initial)	4.515	0.019[2]	0.699061[3]
Bulk Earth (initial)	4.515	0.0897	0.699061
Bulk Earth (modern upper limit)	0	0.0897	0.7049
Depleted mantle (earliest initiation)	3.8	–	0.69996
Depleted mantle (modern)	0	–	0.70263 ± 44[4]

Supplementary Table 1: Summary of the values used to model the strontium isotope evolution of the Earth and Moon (Fig. 4c). Values for the modern bulk Earth and the depleted mantle at 3.8 Ga represent the estimated maximum values determined in this study.

Supplementary Table 2

Parameter	Value
RF power	1250 W
Coolant gas flow rate	16 L/min
Auxiliary gas flow rate	0.85 L/min
Nebuliser gas flow rate	1.3 L/min
Sample uptake rate	200 µL/min
Integration time per isotope	0.02-0.03 s
Total measurement duration	41 s
Washout time	120 s

Supplementary Table 2: SF-ICP-MS operating conditions and method settings during solution Rb/Sr and K/Ca measurement.

Supplementary Table 3

Element	LOD (ppt)
K	1,351
Ca	702
Rb	0.99
Sr	1.8

Supplementary Table 3: calculated limits of detection (LOD) for each element during solution K/Ca and Rb/Sr measurement.

Supplementary Table 4

Element	Isotope	Abundance (%)	Isotopic Mass (u)	Atomic Weight (u)
K	^{39}K	93.2581	38.96370668	39.0983 ± 0.0001
	^{41}K	6.7302	40.96182526	
Ca	^{40}Ca	96.941	39.96259098	40.078 ± 0.004
	^{42}Ca	0.647	41.95861801	
	^{43}Ca	0.135	42.95876644	
	^{44}Ca	2.086	43.95548156	
Rb	^{85}Rb	72.17	84.911789738	85.4678 ± 0.0003
	^{87}Rb	27.83	86.909180531	
Sr	^{84}Sr	0.56	83.9134191	87.62 ± 0.01
	^{86}Sr	9.86	85.9092606	
	^{87}Sr	7.00	86.9088775	
	^{88}Sr	82.58	87.9056125	

Supplementary Table 4: Relative atomic masses, natural isotopic abundances, and atomic weights used in this study.

Supplementary Figure 1: Windimurra Igneous Complex samples

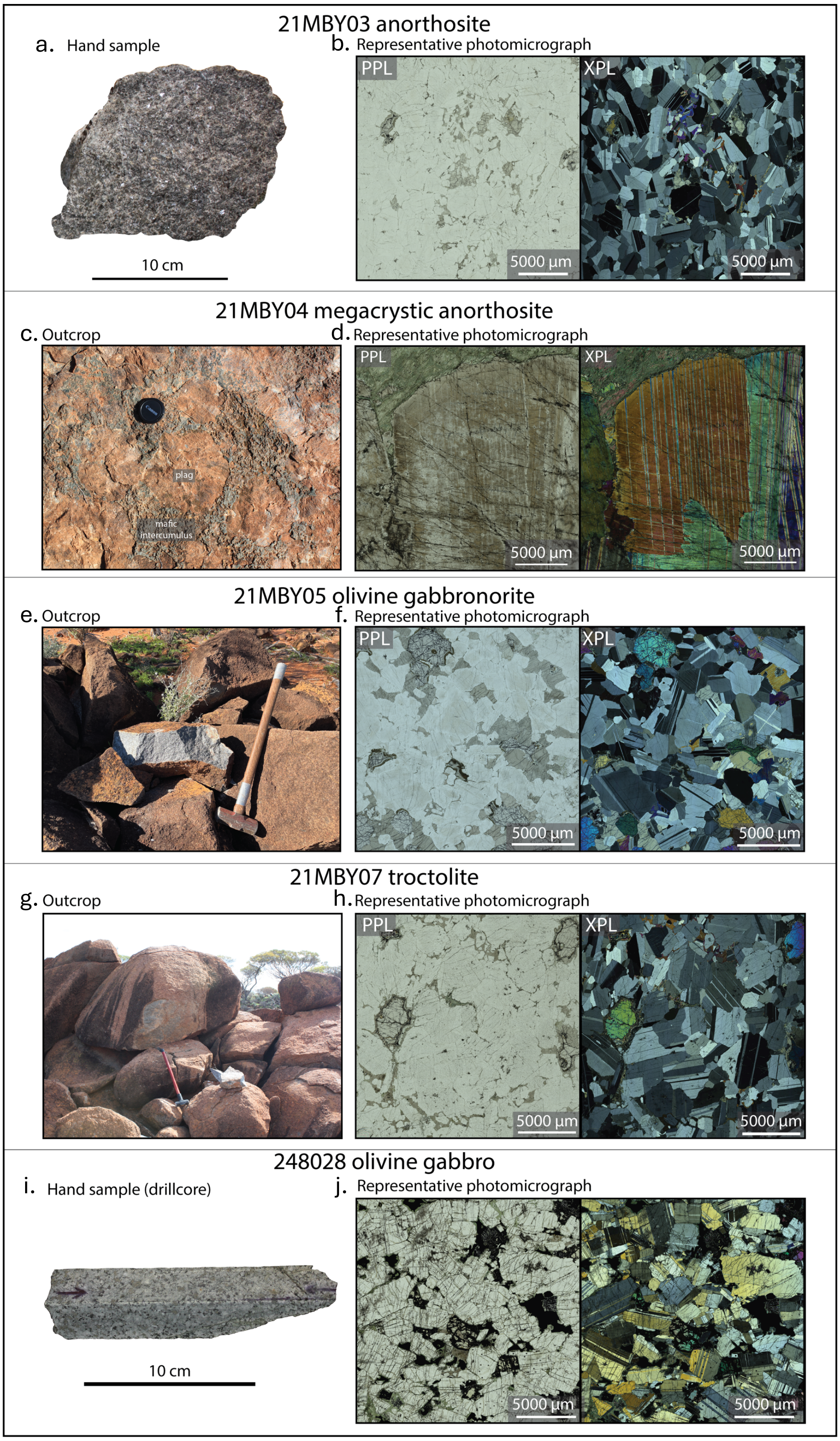

Supplementary Figure 1: Representative outcrop and hand sample photographs and photomicrographs of Windimurra Igneous Complex samples analysed in this study. a) Medium-grained anorthosite 21MBY03 in hand specimen. b) Optical image (ppl, xpl) of 21MBY03. Plagioclase and all mafic phases are in pristine condition, with all magmatic textures and minerals preserved. c) Megacrystic anorthosite 21MBY04 in outcrop. Megacrysts consist of agglomerates of large (~5-10cm) tabular crystals, rather than equant single crystals. d) Optical image (ppl, xpl) of 21MBY04. Plagioclase is optically dark grey in colour and is inclusion rich. e) Olivine gabbronorite 21MBY05 in outcrop. f) Optical image (ppl, xpl) of 21MBY05. Plagioclase and all mafic phases are in pristine condition. g) Troctolite 21MBY07 in outcrop. h) Optical image (ppl, xpl) of 21MBY07. Plagioclase and all mafic phases are very fresh. i) Olivine gabbro 248028 in hand sample. j) Optical image (ppl, xpl) of 248028. This sample is magnetite-bearing.

Supplementary Figure 2: Fiskenæsset Complex samples

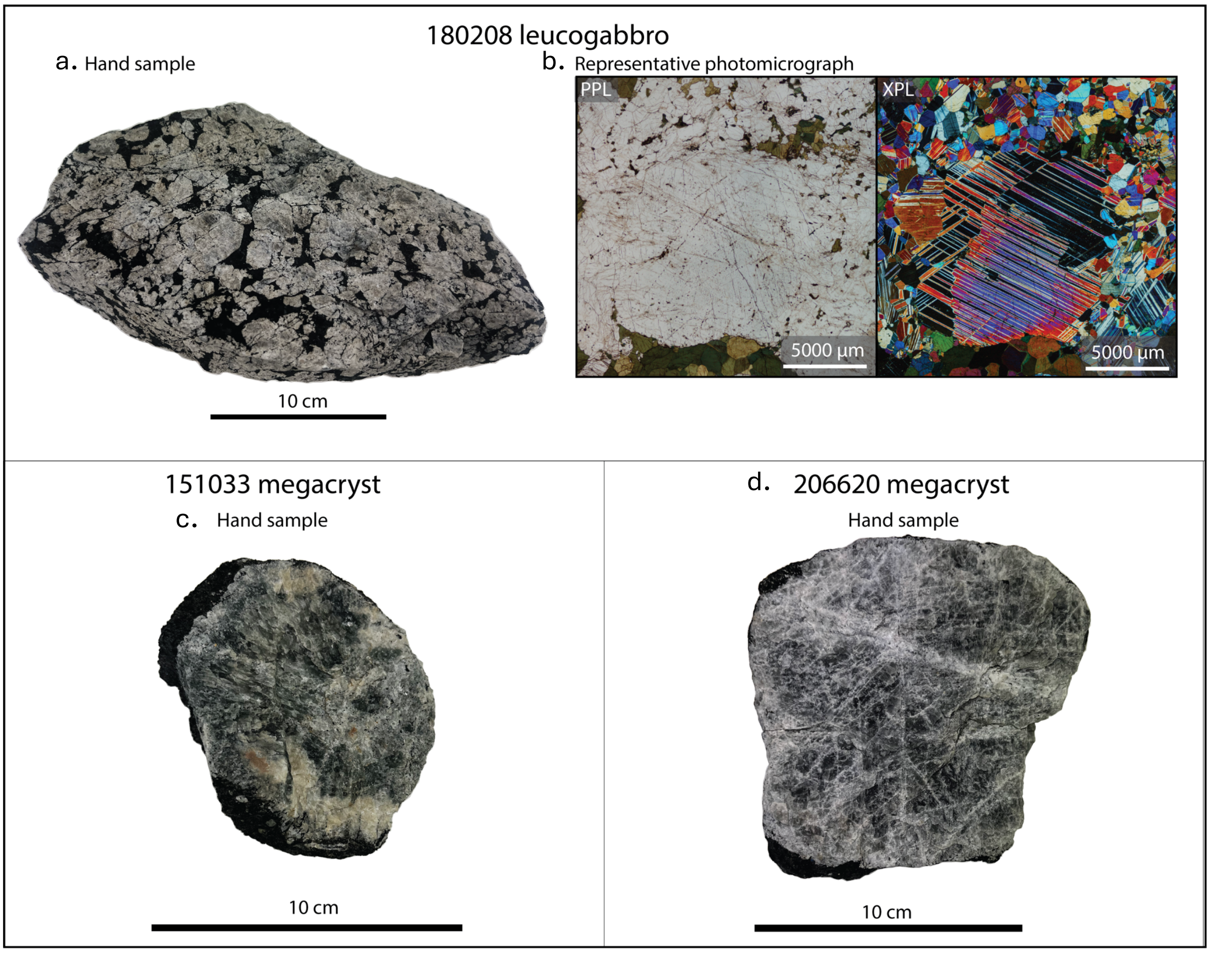

Supplementary Figure 2. a-b) Representative hand sample photographs and photomicrograph (of thick section 180208) of the Fiskenæsset Complex samples analysed in this study. Plagioclase is partially recrystallised. c-d) Hand sample images of plagioclase megacrysts 151033 and 206620. No photomicrographs are included of samples 151033 and 206620, as only polished mounts were prepared of these samples.

Supplementary Figure 3: Manfred Complex samples

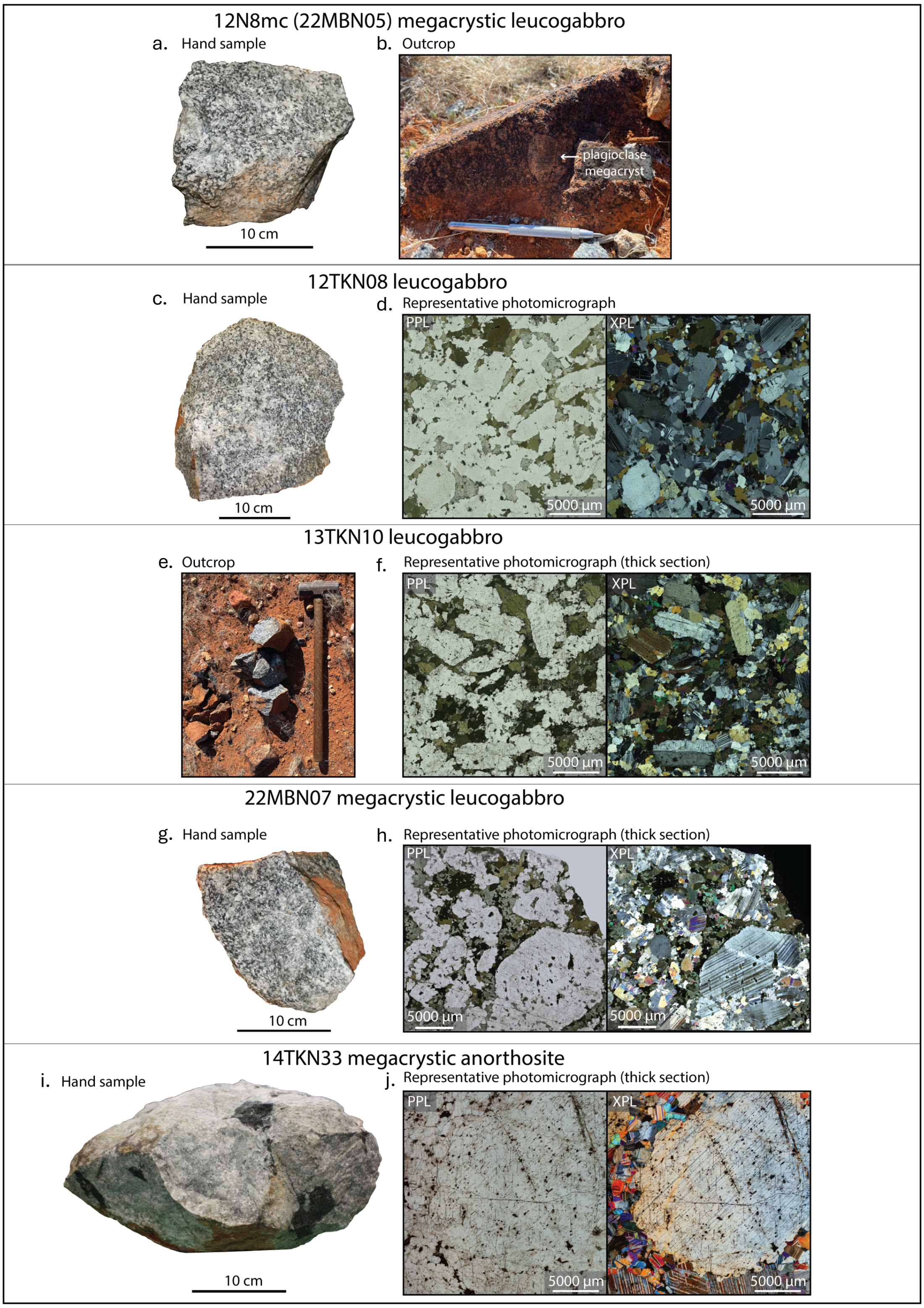

Supplementary Figure 3. Representative outcrop and hand sample photographs and photomicrographs of Manfred Complex anorthosites and leucogabbros analysed in this study. a) Megacrystic leucogabbro 12N8mc/22MBN05 in hand specimen, the sample analysed for LA-MC-

ICPMS Sr mapping in Fig. 2. b) Megacrystic leucogabbro 12N8mc in outcrop. Plagioclase megacrysts are visible on the weathered surface of the rock. c) Leucogabbro 12TKN08 in hand specimen. d) Optical images (ppl, xpl) of leucogabbro 12TKN08. Plagioclase is medium-grained and locally retains magmatic textures. Intercumulus magmatic clinopyroxene is partially replaced by hornblende. e) Leucogabbro 13TKN10 was collected from low-lying outcrop. f) Optical images (ppl, xpl) of leucogabbro 13TKN10. Plagioclase is partially altered, locally retaining magmatic textures. g) Megacrystic leucogabbro 22MBN07 in hand specimen. h) Optical images (ppl, xpl) of megacrystic leucogabbro 22MBN07. Magmatic mineralogy and textures are partially preserved. The large megacryst visible is the same crystal from Fig. 2. i) Megacrystic anorthosite 14TKN33 in hand specimen. Single equant crystals (now mostly recrystallised) of plagioclase are as large as 10cm. j) Optical image (ppl, xpl) of 14TKN33. Plagioclase is partially recrystallised and altered.

Supplementary Figure 4: Fiskenæsset Complex megacryst micro-milling

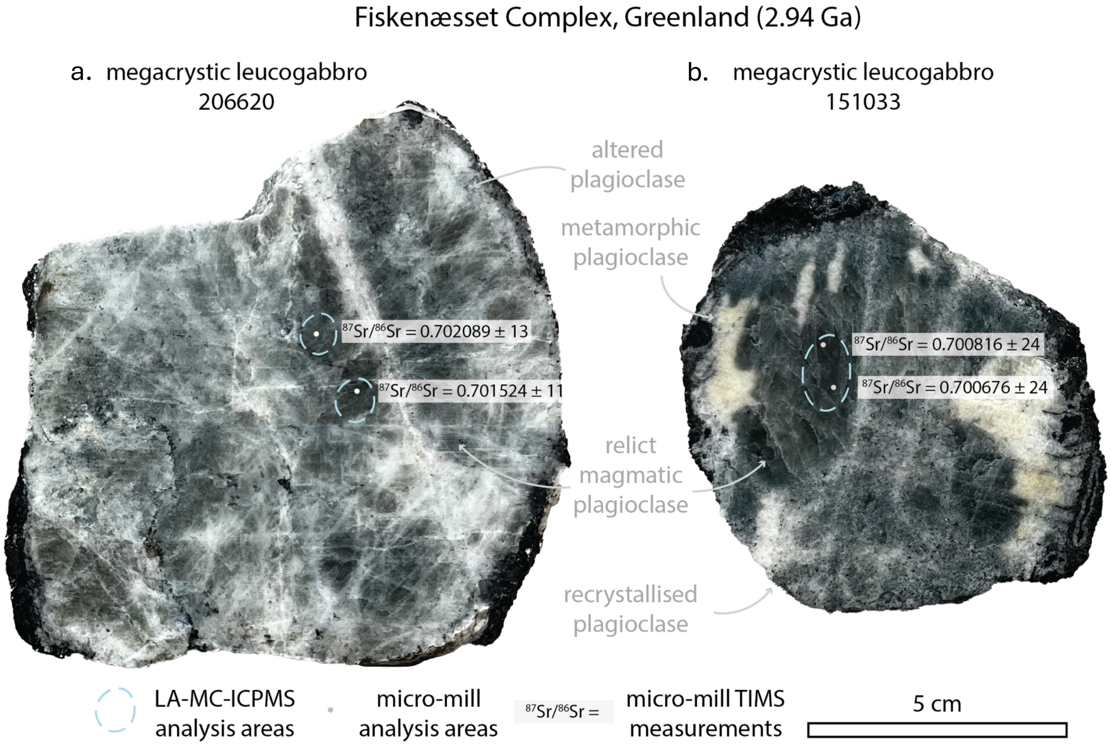

Supplementary Figure 4. Areas targeted for LA-MC-ICPMS and micro-milling from Fiskenæsset Complex plagioclase megacrysts (a) 206620 and (b) 151033. Domains outlined by blue dotted lines are the domains drilled from the sample, mounted in epoxy resin and analysed by LA-MC-ICPMS point analyses. Micro-milled areas target the least radiogenic LA-MC-ICPMS measurements. Measured TIMS results (without age corrections applied) for each milled area are displayed in the grey boxes.

Supplementary Figure 5: Manfred Complex 12N8mc (22MBN05) micro-mill targets

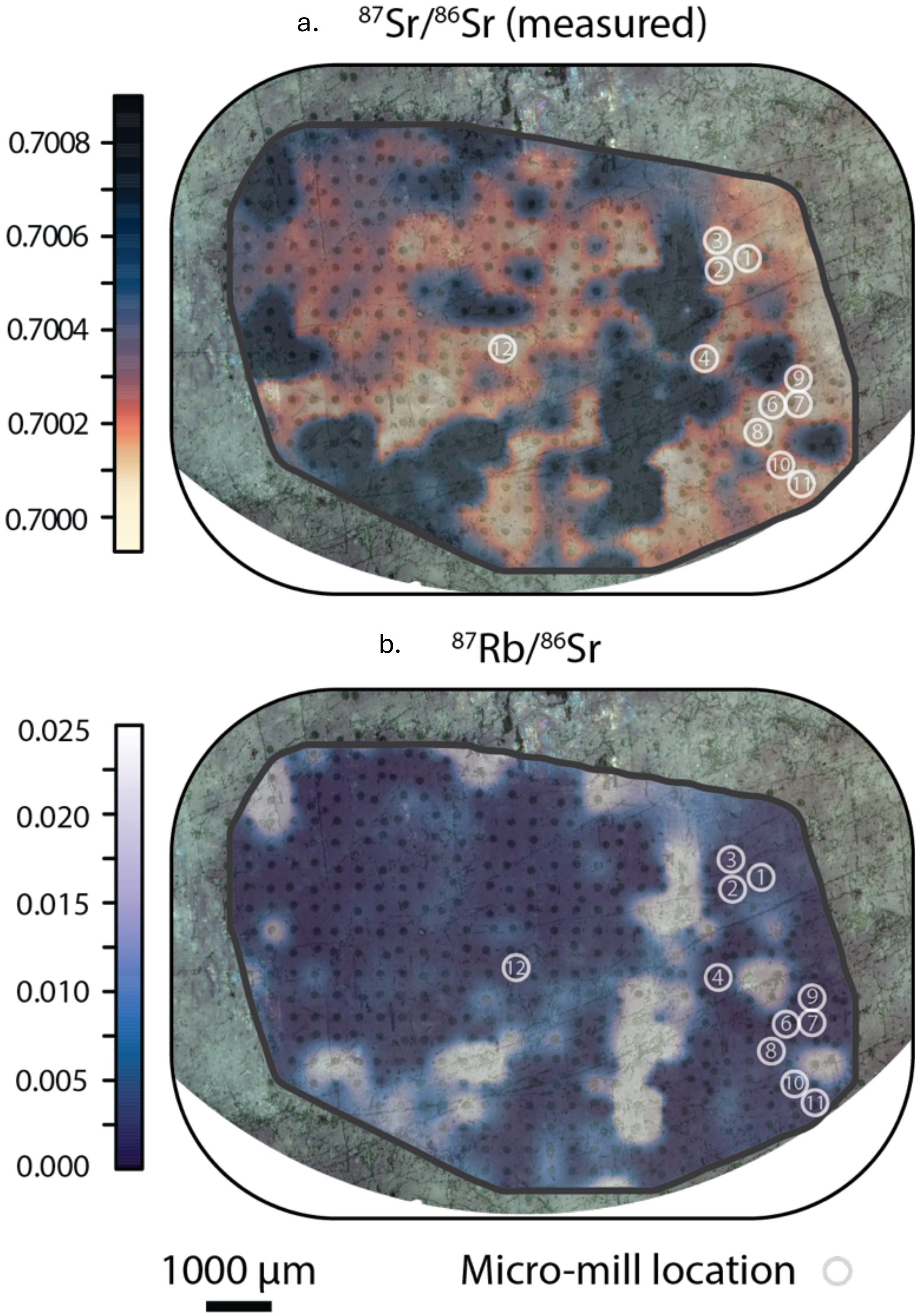

Supplementary Figure 5. Areas of Manfred Complex megacryst 12N8mc targeted for micro-milling. Plotted numbers correspond with the aliquot number for each analysis in

Supplementary Tables 4-5 (as analysed for TIMS Sr isotopes and Proteus Ca isotopes). a) Measured $^{87}Sr/^{86}Sr$, with no age correction applied. b) Measured $^{87}Rb/^{86}Sr$.

Supplementary Figure 6: LA-MC-ICPMS ablations (12N8mc)

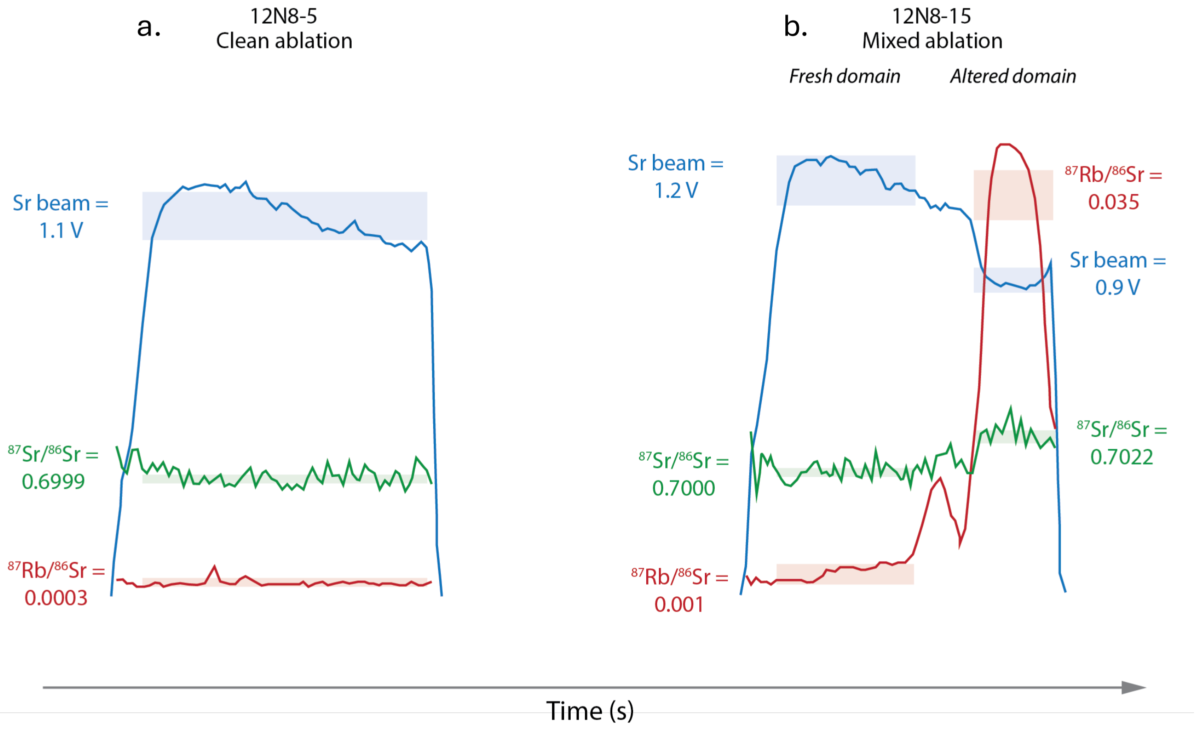

Supplementary Figure 6. Examples of two ablations from Manfred Complex sample 12N8mc. On the left is an example of a clean ablation. In a), the laser intersects an altered domain/inclusion at depth, resulting in a spike in $^{87}Sr/^{86}Sr$, $^{87}Rb/^{86}Sr$, and a drop in the total Sr beam. In ablations such as b), the integration is cropped to avoid incorporating data from the altered domain.

Supplementary Figure 7: Windimurra Igneous Complex (2.81 Ga) LA-MC-ICPMS Sr results

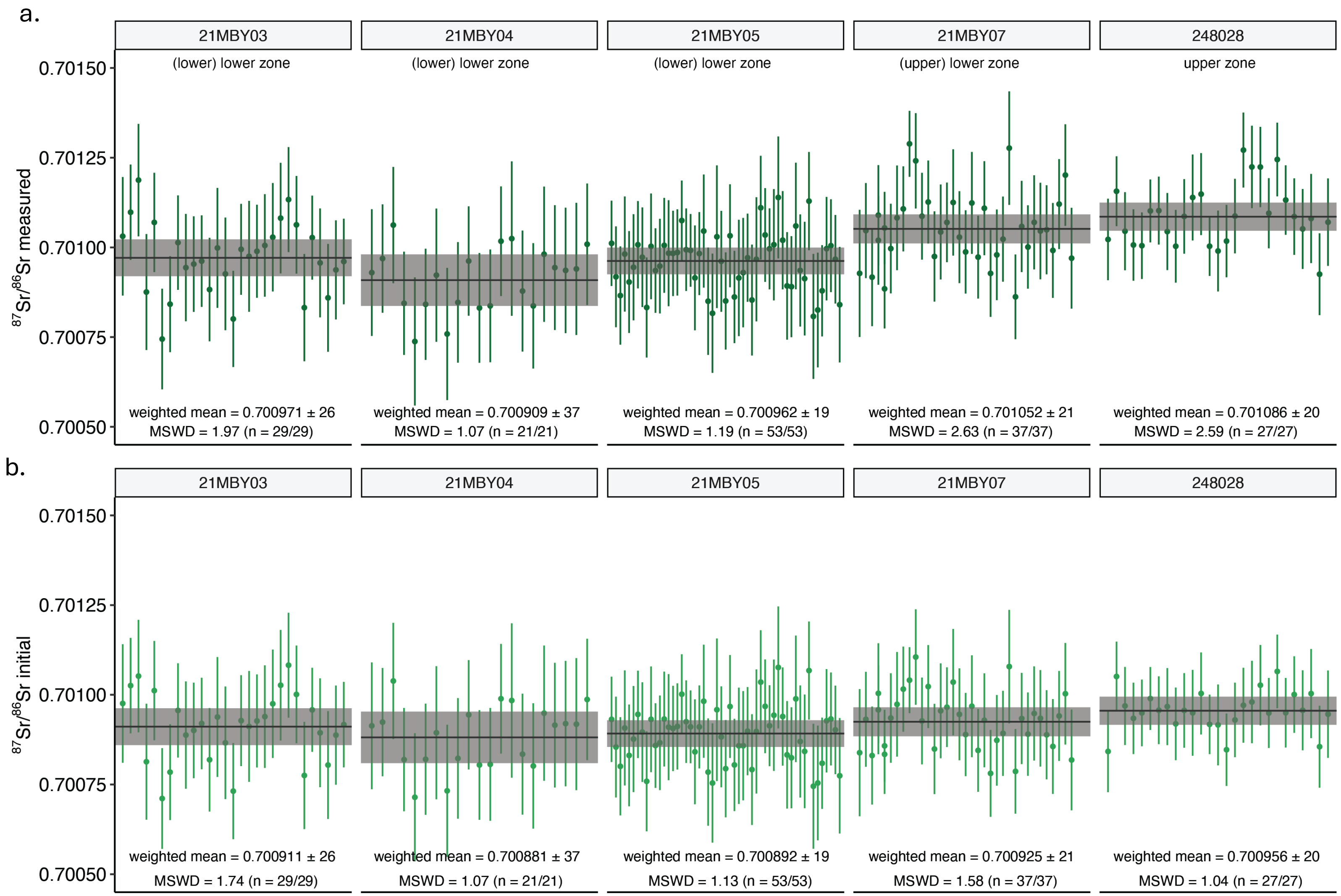

Supplementary Figure 7: All LA-MC-ICPMS analyses for the Windimurra Igneous Complex samples. Measured $^{87}Sr/^{86}Sr$ are shown in a), and age-corrected $^{87}Sr/^{86}Sr$ are shown in b). For each sample, the black line represents the weighted mean of the included analyses, and the grey horizontal band represents the 95% confidence interval. Outliers (>3SD) for $^{87}Sr/^{86}Sr$ and $^{87}Rb/^{86}Sr$ are coloured grey and are excluded from initials and weighted means. All samples have MSWD ≈ 1, indicating that these samples are undisturbed. Measured $^{87}Sr/^{86}Sr$ in samples from multiple areas of the intrusion show good agreement, providing further evidence that these reflect primary $^{87}Sr/^{86}Sr$ values.

Supplementary Figure 8: Fiskenæsset Complex (2.94 Ga) LA-MC-ICPMS Sr results

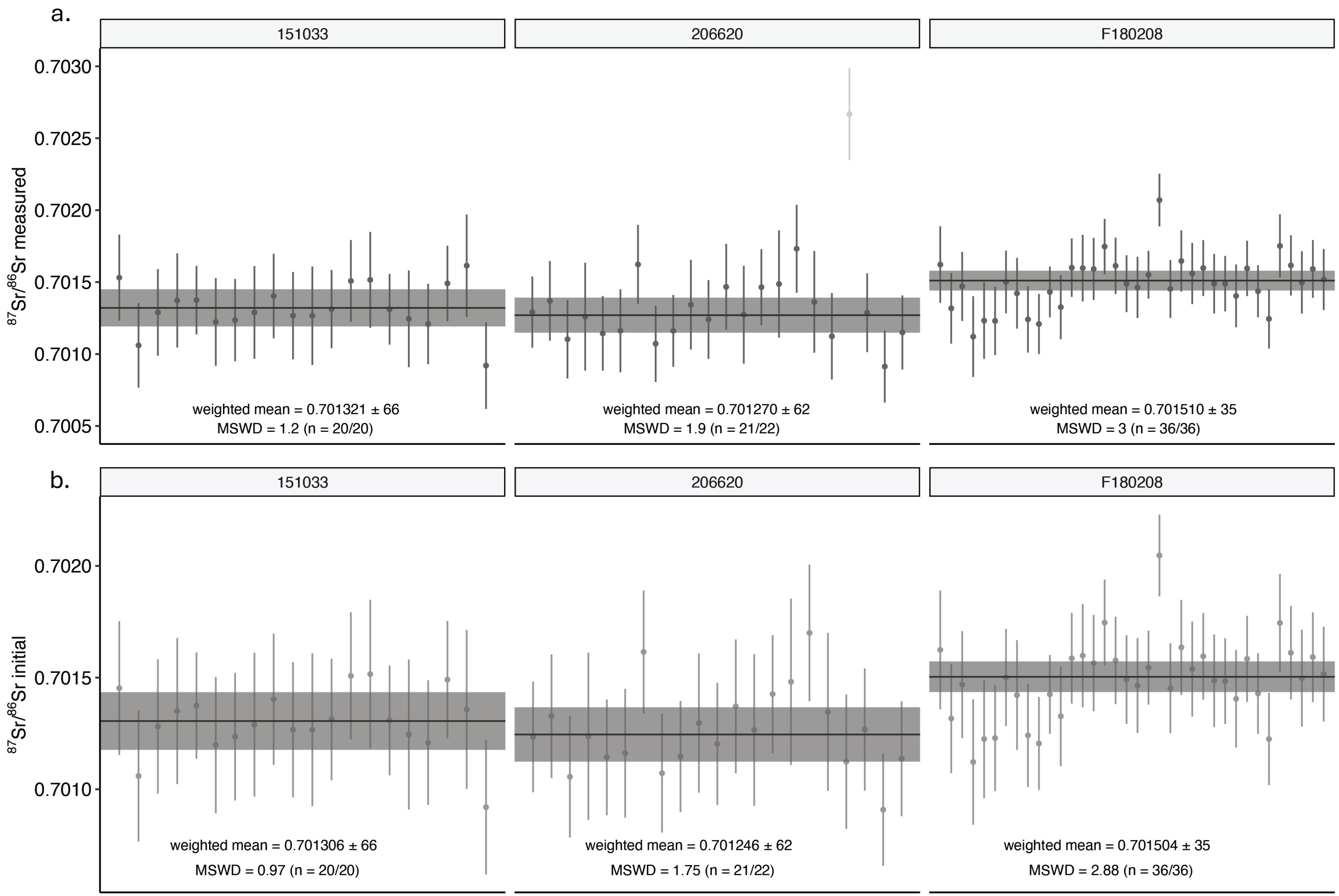

Supplementary Figure 8. All LA-MC-ICPMS analyses for the Fiskenæsset Complex. Measured $^{87}Sr/^{86}Sr$ are shown in a), and age-corrected $^{87}Sr/^{86}Sr$ are shown in b). Outliers (>3SD) for $^{87}Sr/^{86}Sr$ and $^{87}Rb/^{86}Sr$ are coloured in grey and are excluded from initials and weighted means. For each sample, the black line represents the weighted mean of the included analyses, and the grey horizontal band represents the 95% confidence interval. Samples mostly have MSWD ≈ 1, with minor scatter observed. Several measured $^{87}Sr/^{86}Sr$ values are lower than the rest of the population, indicating that there is some heterogeneity, either as a primary magmatic feature or from later disturbance. This is supported by the unradiogenic TIMS measurements (e.g. Supp. Fig. 4).

Supplementary Figure 9: Manfred Complex (3.73 Ga) LA-MC-ICPMS Sr results

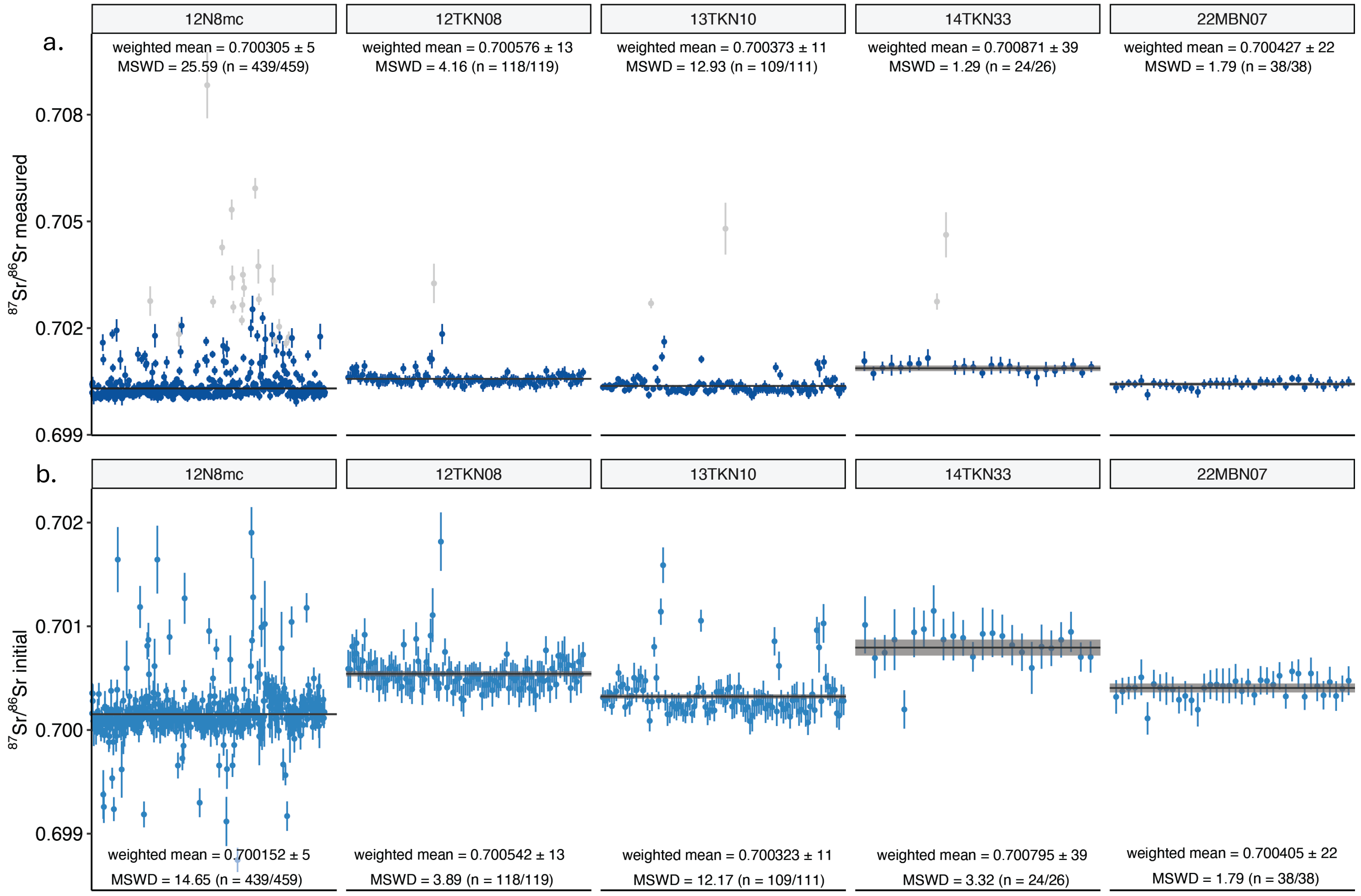

Supplementary Figure 9. All LA-MC-ICPMS analyses for the Manfred Complex samples. Measured $^{87}Sr/^{86}Sr$ are shown in a), and age-corrected $^{87}Sr/^{86}Sr$ are shown in b). Outliers (>3SD) for $^{87}Sr/^{86}Sr$ and $^{87}Rb/^{86}Sr$ are coloured grey and are excluded from initials and weighted means. For each sample, the black line represents the weighted mean of the included analyses, and the grey horizontal band represents the 95% confidence interval. Samples have MSWD >> 1, indicating all are disturbed to some degree. Many samples have populations of unradiogenic measured $^{87}Sr/^{86}Sr$ (<0.7001), indicating that primary values are locally preserved (e.g. Fig. 1). The high scatter in the 12N8mc dataset indicates some Rb/Sr elevated analyses remain despite the outlier rejection, resulting in some overcorrections. Due to partial disturbance, the weighted mean was therefore determined by averaging the least radiogenic (and low Rb/Sr) domains from the LA-MC-ICPMS map (Fig. 2).

Supplementary Figure 10: Calcium isotope measurements

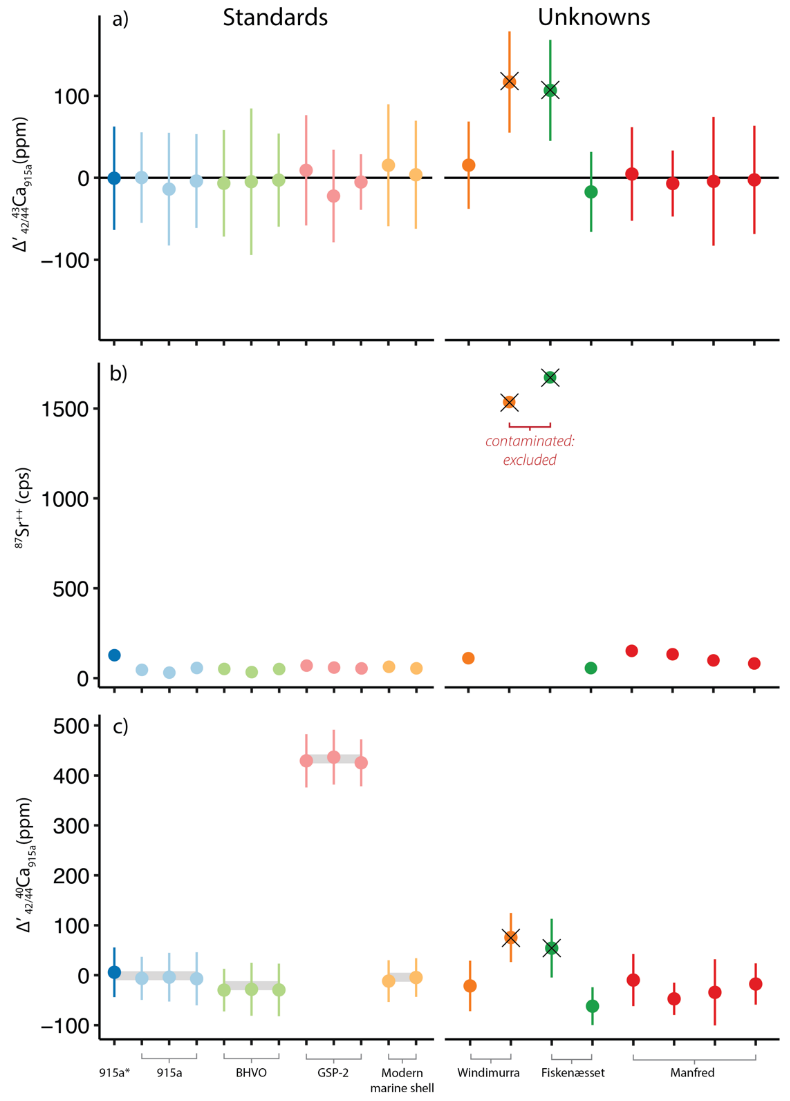

Supplementary Figure 10. Calcium isotope measurements of reference materials and unknowns. Asterisks denote unprocessed reference material solutions. a) Δ′^{43}Ca$_{915a}$ measurements, in ppm. Plotted are the mean and 2SD of each analysis. All analyses, except 04-2 and 15-1, are within uncertainty of zero. b) ^{87}Sr^{++} measurements in counts per second, measured as a monitor of contamination. All measurements are low, except for 04-2 and 15-1, indicating that these two samples are contaminated, and are therefore excluded. c) Δ′^{40}Ca$_{915a}$ measurements, in ppm. Grey bars represent published reference material values[5]. Plotted are the mean and 2SD of each analysis.

Supplementary Figure 11: Micro-mill TIMS strontium isotope measurements

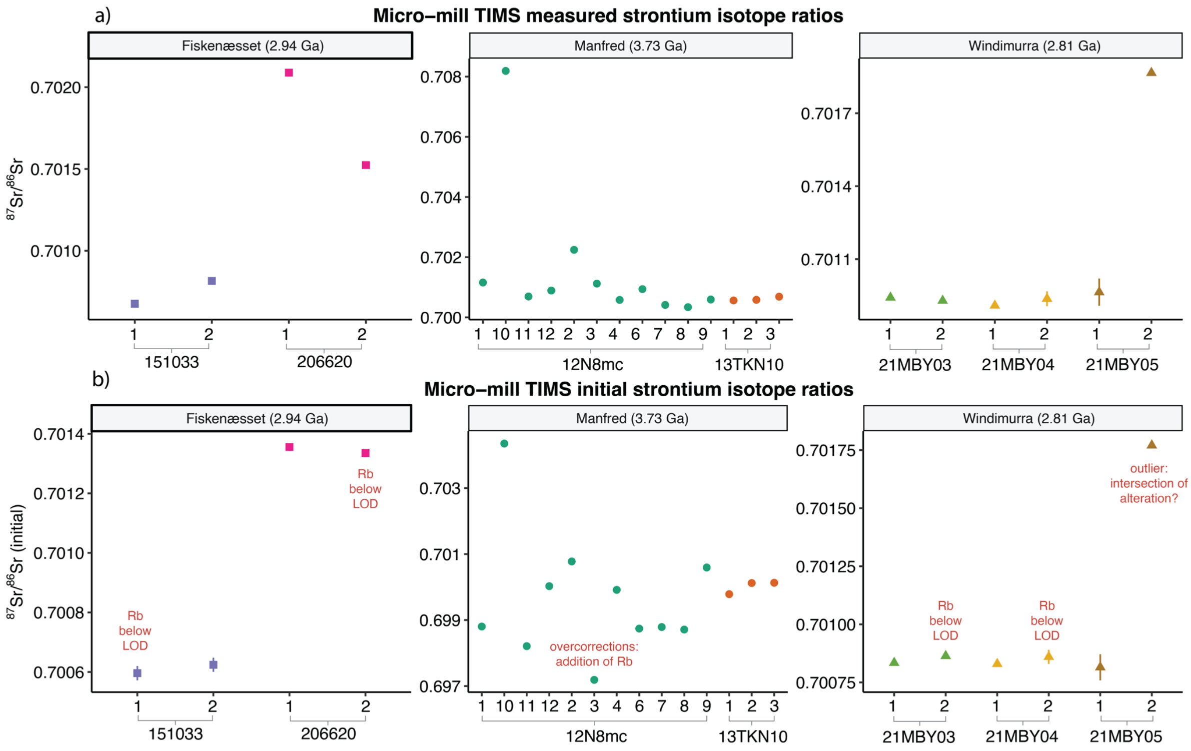

Supplementary Figure 11. Micro-mill TIMS strontium isotope measurements of all samples. All uncertainties plotted as 2SE. a) Measured ratios. b) Initial ratios. The Fiskenæsset and Windimurra analyses, comparatively better preserved than the Manfred Complex, show less scatter and are mostly close to LA-MC-ICPMS results. TIMS analyses of Fiskenæsset sample 151033 are notably less radiogenic than the LA-MC-ICPMS average, but are within uncertainty of the least radiogenic individual laser analyses. This may be due to the difference in spatial resolution and/or sample heterogeneity, with the micro-mill sampling a fresher domain at depth. Manfred Complex analyses are scattered, with some analyses overcorrected to unrealistically unradiogenic initial ratios due to later addition of Rb.

References

1. Hans, U., Kleine, T. & Bourdon, B. Rb–Sr chronology of volatile depletion in differentiated protoplanets: BABI, ADOR and ALL revisited. *Earth and Planetary Science Letters* **374**, 204–214 (2013).
2. Borg, L. E., Brennecka, G. A. & Kruijer, T. S. The origin of volatile elements in the Earth–Moon system. *Proc. Natl. Acad. Sci. U.S.A.* **119**, e2115726119 (2022).
3. Yobregat, E., Fitoussi, C. & Bourdon, B. Rb-Sr constraints on the age of Moon formation. *Icarus* **420**, 116164 (2024).
4. Workman, R. K. & Hart, S. R. Major and trace element composition of the depleted MORB mantle (DMM). *Earth and Planetary Science Letters* **231**, 53–72 (2005).
5. Lewis, J. *et al.* Collision course; high-precision mass-independent and mass-dependent calcium isotope measurements using the prototype collision cell MC-ICPMS/MS, Proteus. *Chemical Geology* **614**, 121185 (2022).
